# Supplementary material for: Cross-sectional and longitudinal changes in category-selectivity in visual cortex following pediatric cortical resection
Source: bioRxiv. 2024 Dec 12:2024.12.08.627367. Preprint. [Version 1] doi: 10.1101/2024.12.08.627367 (PMC11661110; doi:10.1101/2024.12.08.627367)
Supplement: Supplement 1 [file media-1.pdf]

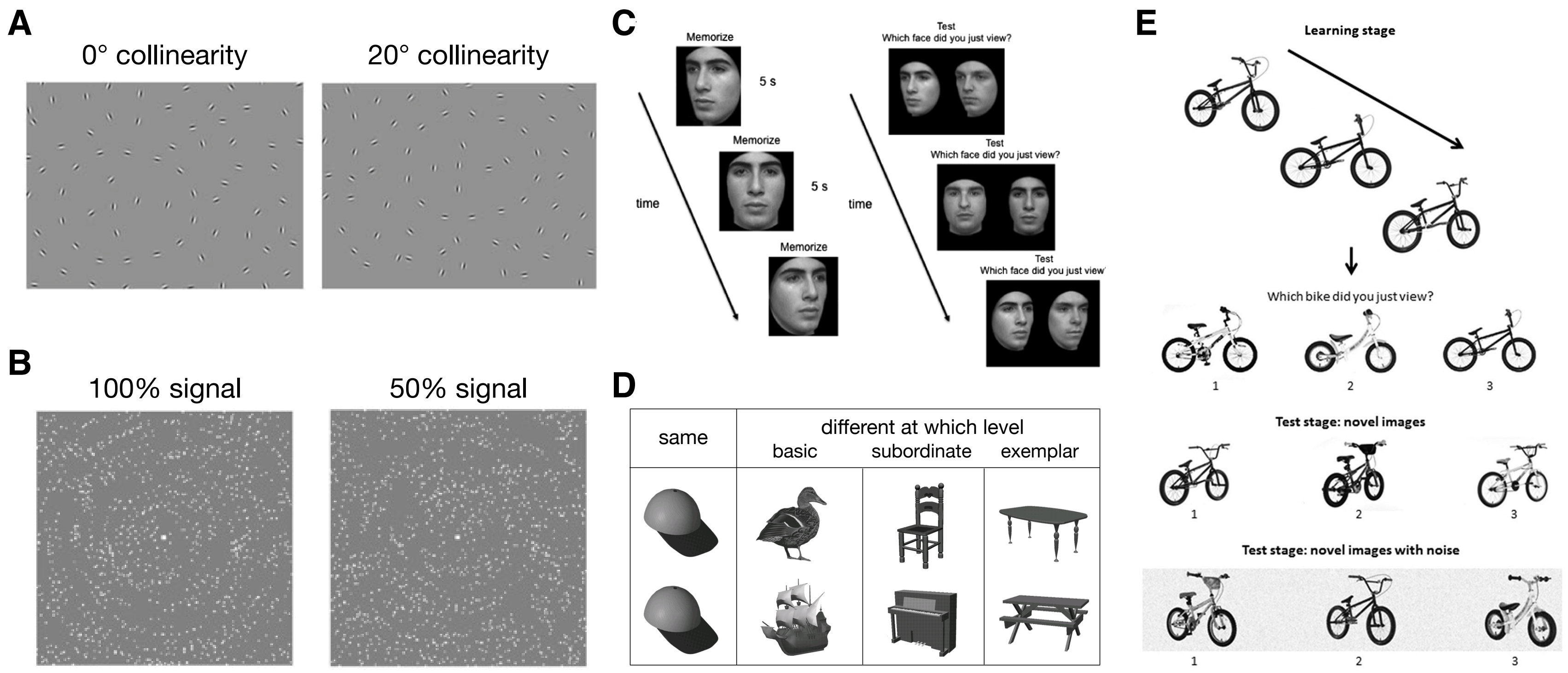

Fig. S1. Design of behavioral experiments.
(**A**) Contour integration (Hadad et al., 2010). Task: Participants viewed a brief presentation of either aligned or misaligned Gabor patches (in separate blocks) and indicated the leftward or rightward direction of the embedded 'egg-like' shape. (**B**) Glass pattern (Lewis et al., 2002). Task: Participants indicated which of the two displays had a more concentric swirl. (**C**) Cambridge Face Memory Test for Children (Croydon et al., 2014). Task: Participants were instructed to remember target faces and subsequently identify them amongst an array of distractor faces. (**D**) Object matching experiment (Gauthier et al., 1999). Task: Participants made same/different discriminations on pairs of objects and pressed a 'same' or 'different' button to indicate their response. (**E**) Cambridge Bicycle Memory Test for Children (Bennetts et al., 2017). Task: Participants were instructed to remember target bicycles and subsequently identify them amongst novel images of bicycles.

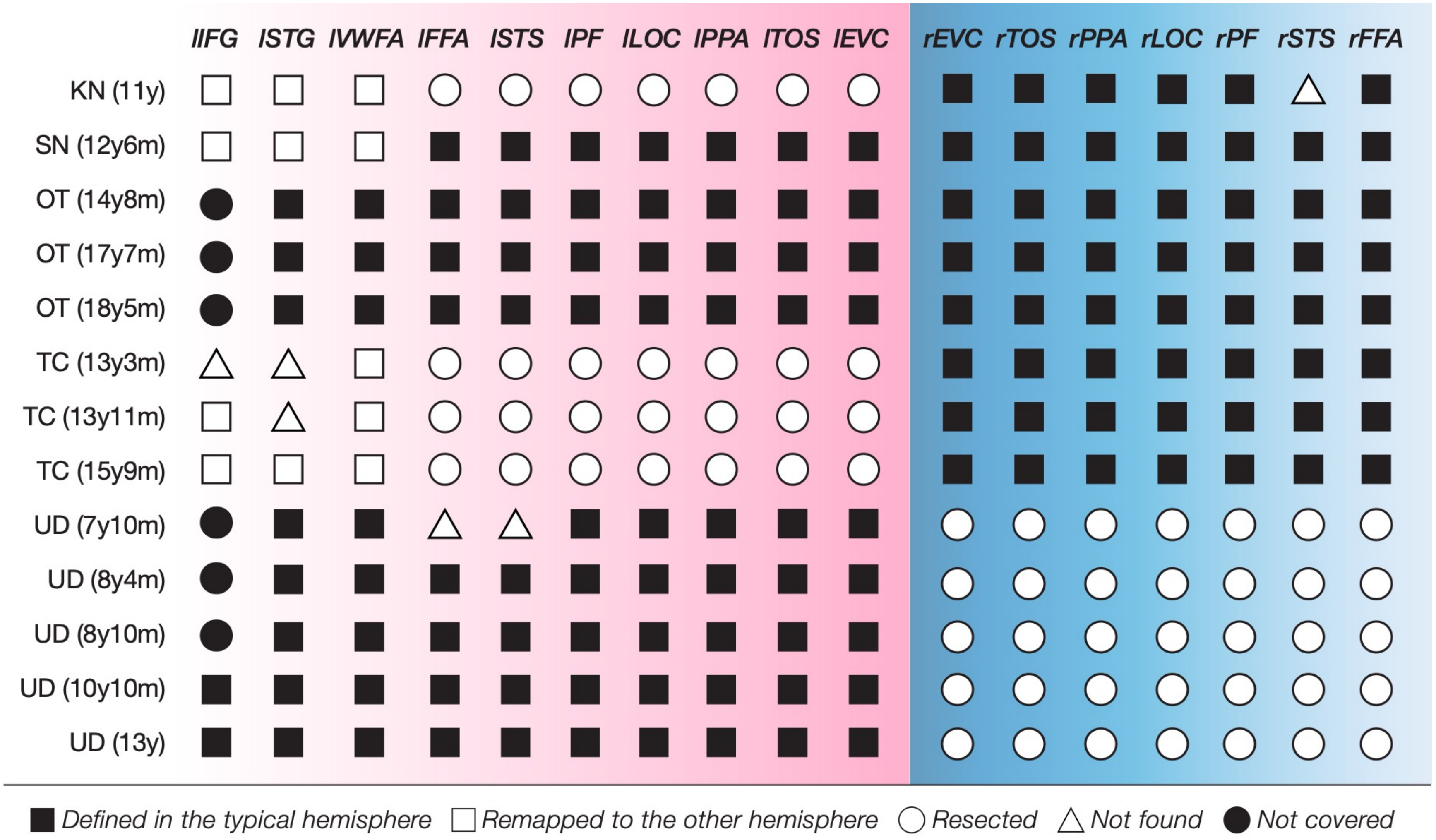

Fig. S2. The presence of category-selective regions of interest (ROIs) in each patient.
l = left, r = right, IFG = inferior frontal gyrus, STG = superior temporal gyrus, VWFA = visual word form area, FFA = fusiform face area, STS = superior temporal sulcus, PF = posterior fusiform, LOC = lateral occipital complex, PPA = parahippocampal place area, TOS = transverse occipital sulcus, EVC = early visual cortex.

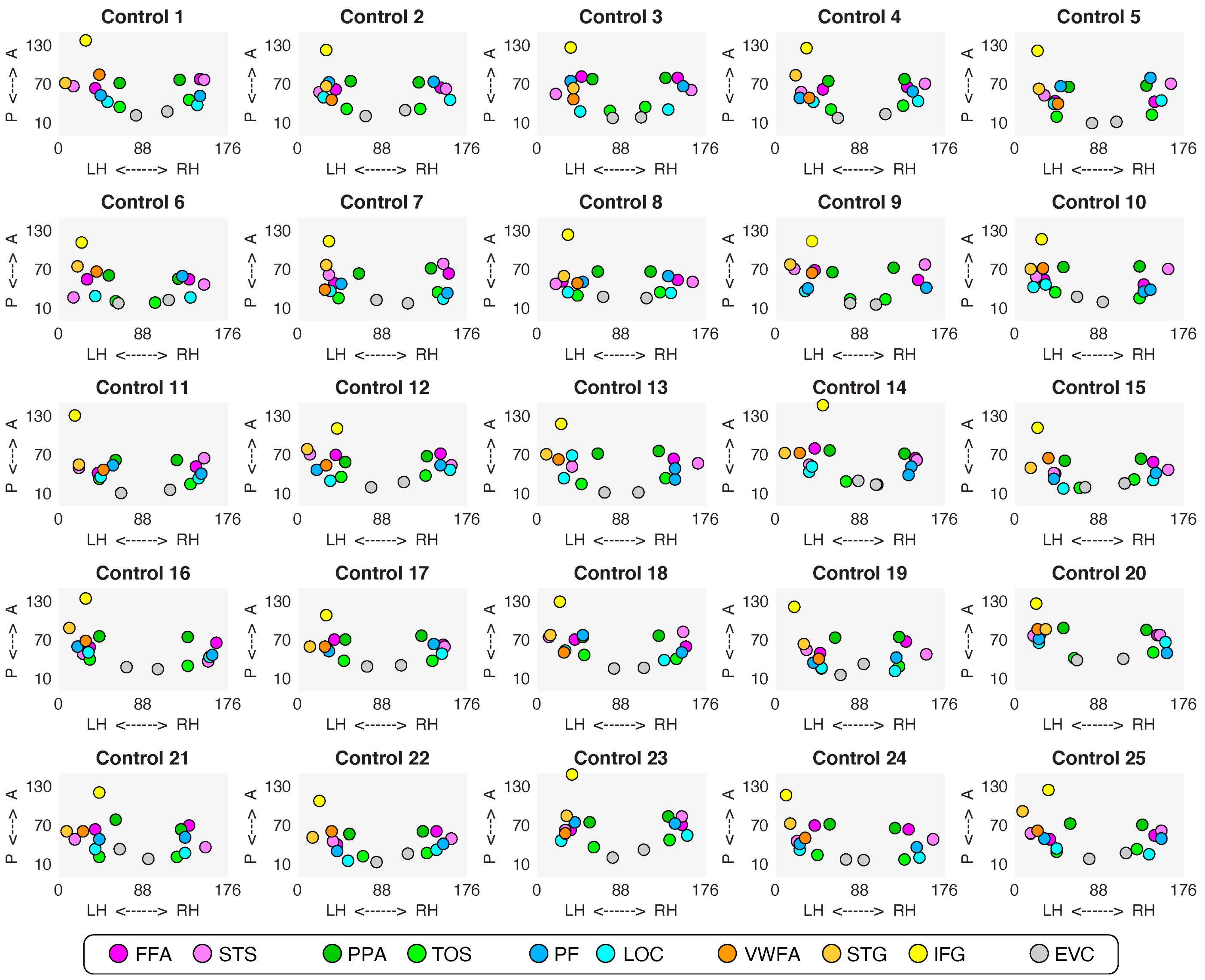

Fig. S3. Spatial organization of category selectivity in each individual control (n=25).

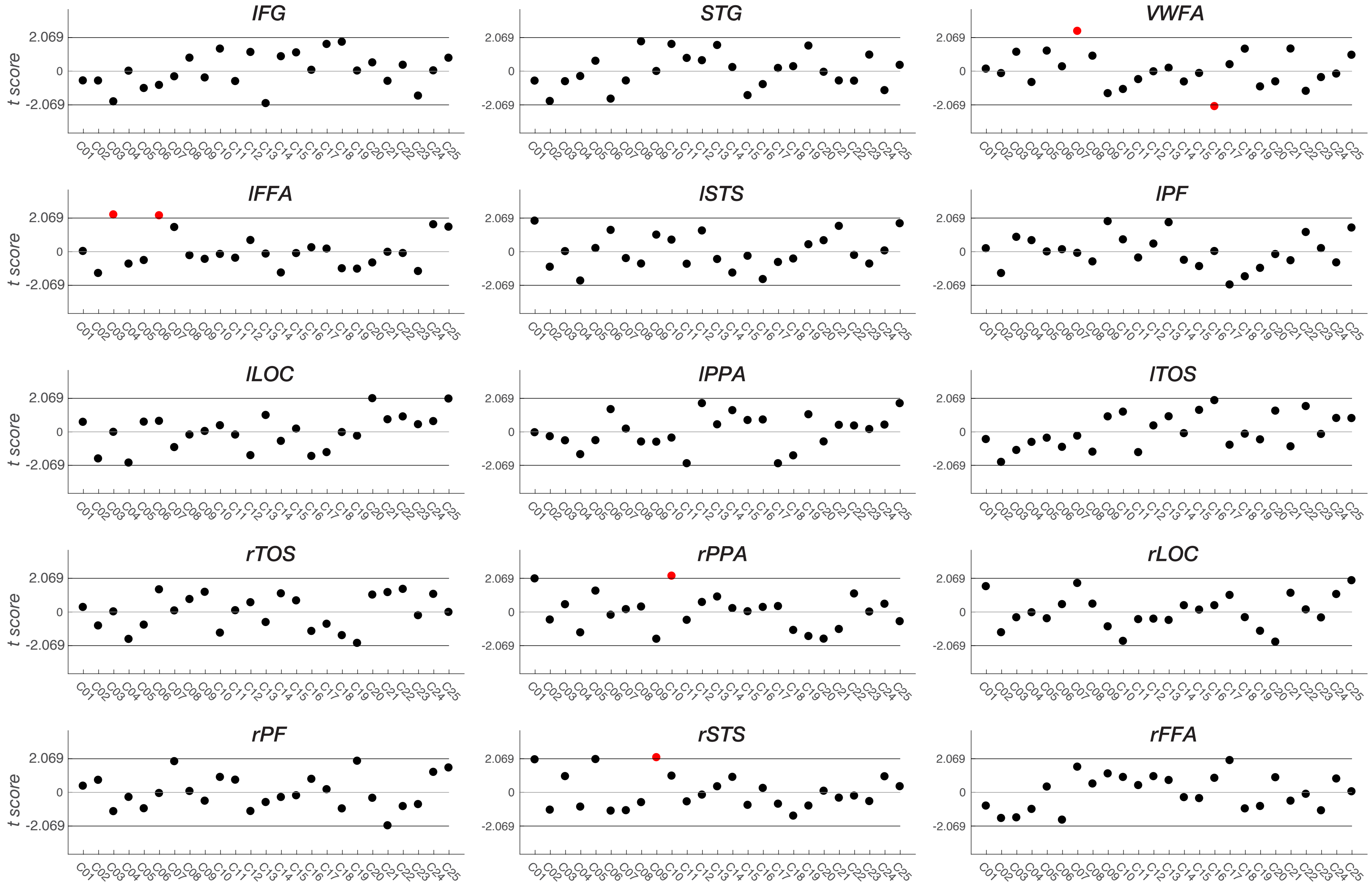

Fig. S4. Crawford's t tests compared representational similarity of all category-selective ROIs in each control participant to the rest of the controls. Red dots indicate significant deviations.

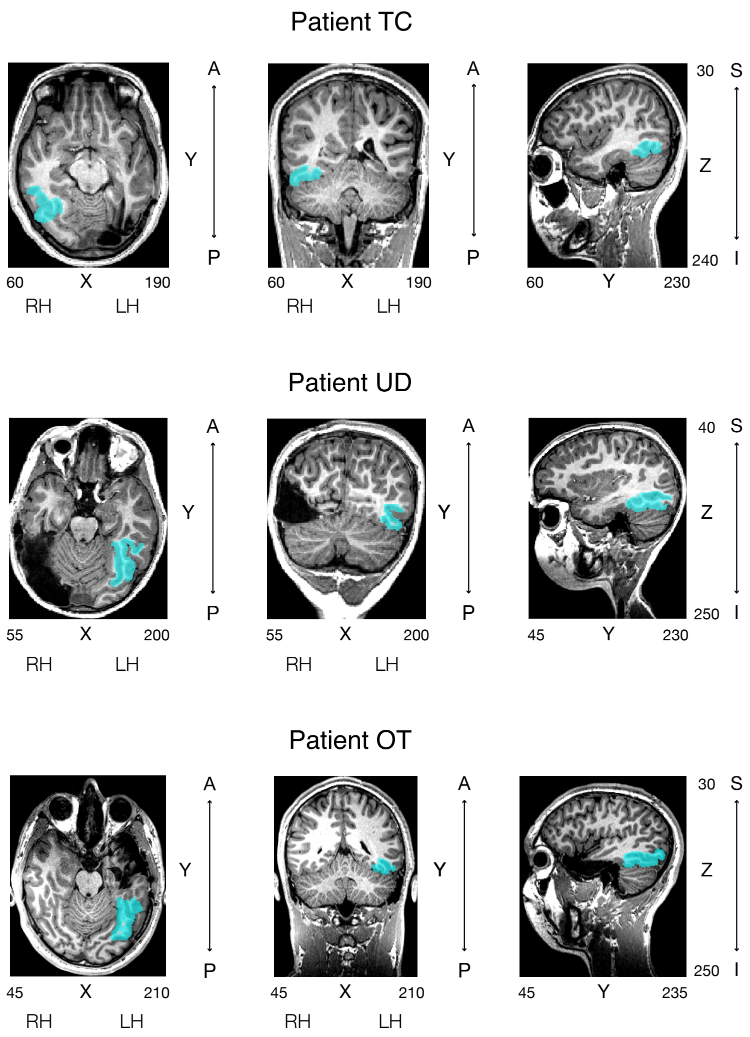

Fig. S5. The anatomically defined right FG/OTS region visualized in corresponding volume space for TC, UD and OT. Total number of anatomical voxels (1mm isotropic) is 7307 in TC, 12428 in UD, and 12013 in OT.
A = anterior, P = posterior, S = superior, I = inferior, RH = right hemisphere, LH = left hemisphere.

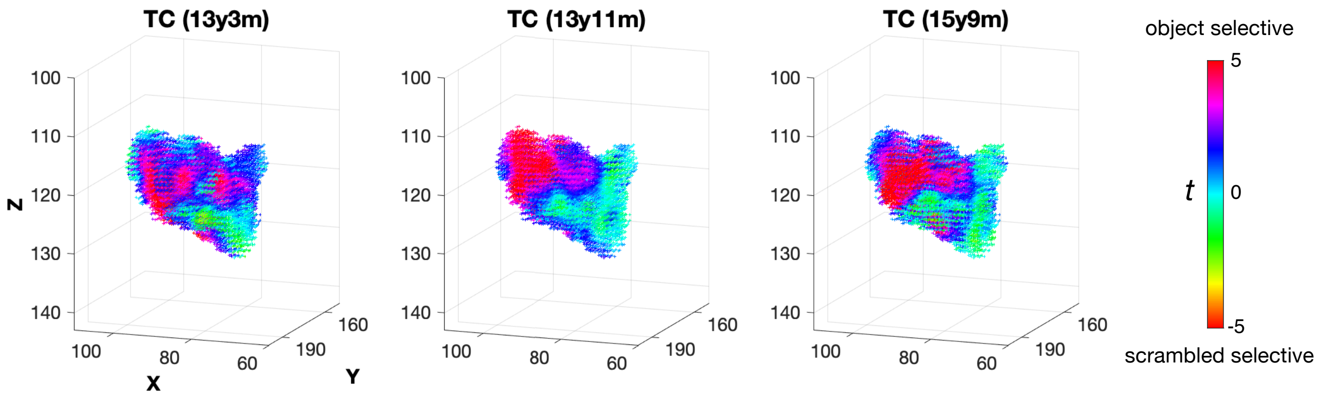

Fig. S6. Longitudinal object versus scramble object selectivity in the anatomically defined FG/OTS in TC (3 sessions).
t score denotes changes over time in each voxel's selectivity of objects over scrambled objects. There were no significant differences between any two scan sessions, all $|t|$ values < 1.549, all p values > 0.122, independent-samples t tests at the voxel level.

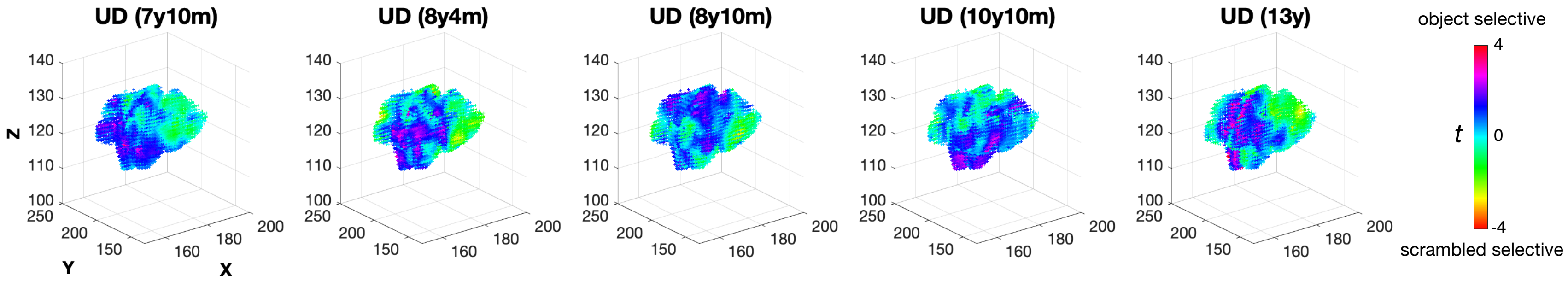

Fig. S7. Longitudinal object versus scramble object selectivity in the anatomically defined FG/OTS in UD (5 sessions*).*
t score denotes changes over time in each voxel's selectivity of objects over scrambled objects. There were no significant differences between any two scan sessions (all $|t|$ values < 1.741, all p values > 0.082), independent-samples t tests at the voxel level.

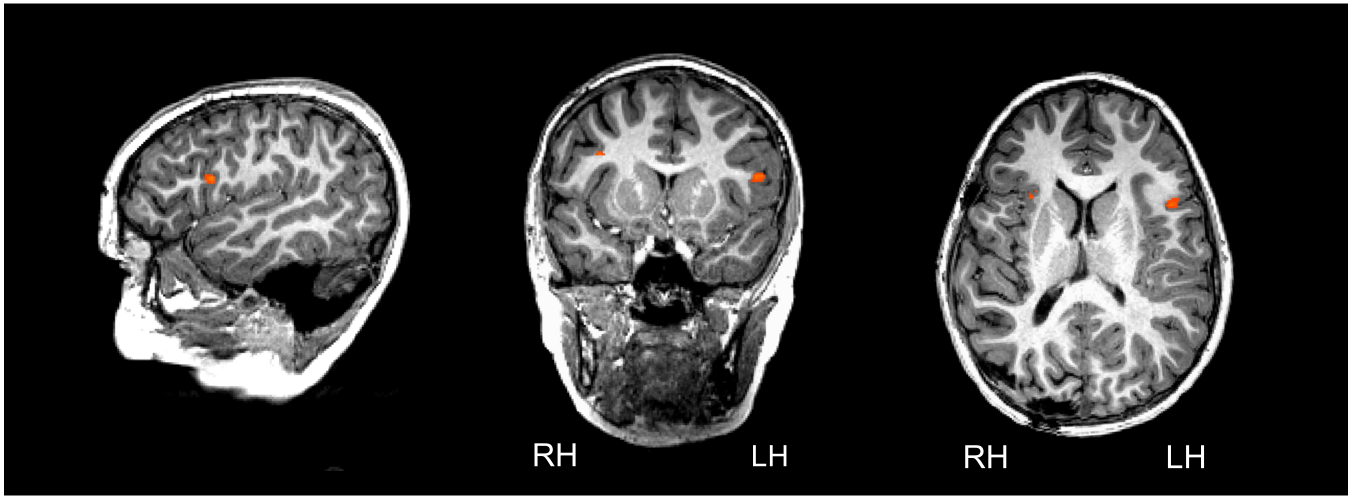

Fig. S8. Post-surgical language mapping results in TC.
Language-selective activation was found in the left Inferior Frontal Gyrus (Broca's area); no language-selective activation was found near the left Superior Temporal Gyrus (Wernicke's area) and no obvious language-selective activation was visible in the right hemisphere.

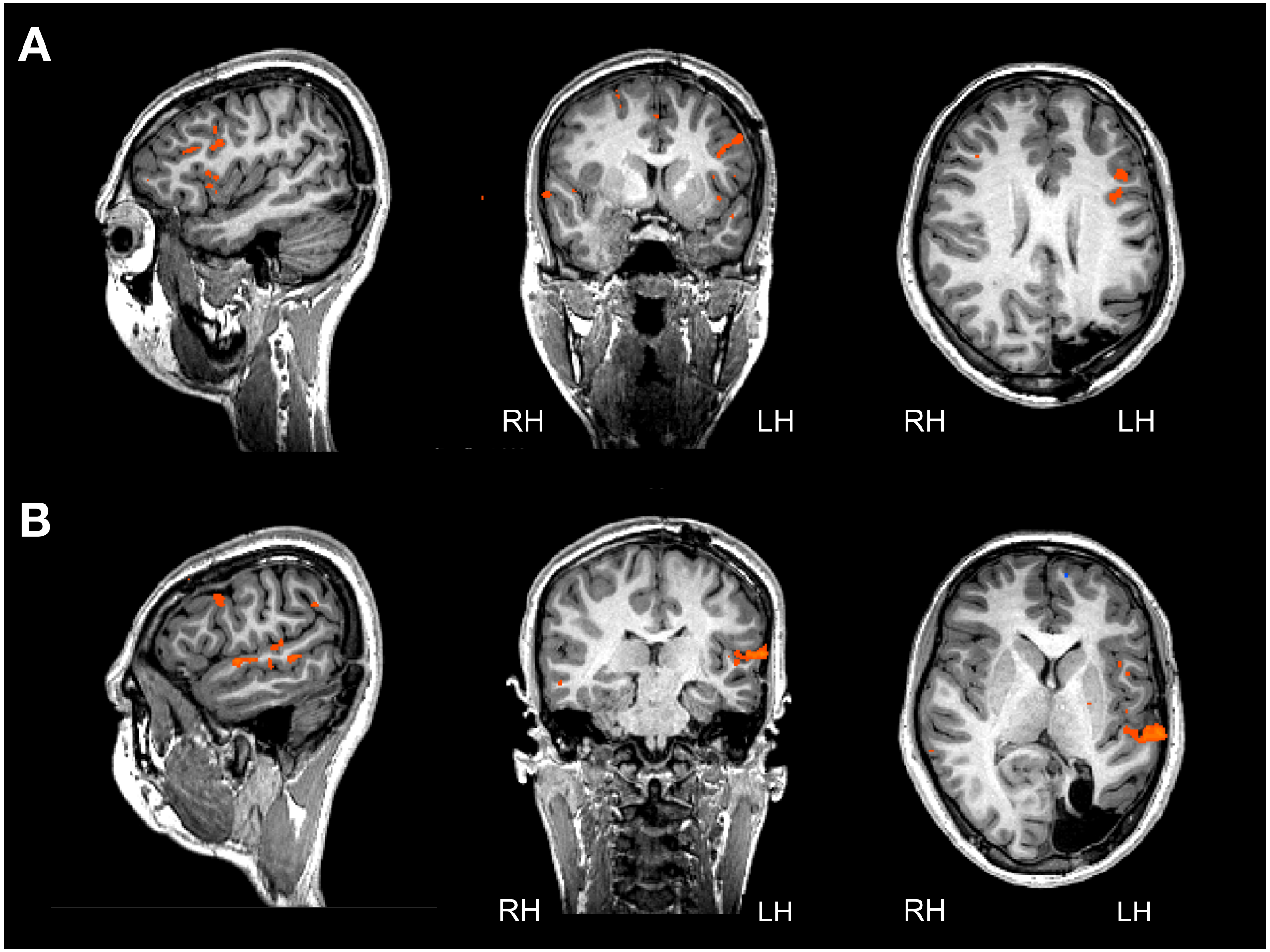

Fig. S9. Post-surgical language mapping results in UD.
A) language-selective activation in the left Inferior Frontal Gyrus (Broca's area)
B) language-selective activation in the left Superior Temporal Gyrus (Wernicke's area)

Table S1. Demographic and surgical information for each of the five patients

Code	Gender	Diagnosis/Pathology	Preop MRI	Age at surgery	Lesioned hemisphere	Resection	Visual Fields	EESOS*
KN	M	N/A	N/A	20m	left	left hemispherectomy	N/A	N/A
SN	M	N/A	N/A	1d	left	evacuation of left temporal hematoma	N/A	N/A
TC	F	perinatal stroke with medically intractable focal epilepsy/multifocal encephalomalacia consistent with remote ischemic injury	abnormal MRI with lesion in the left occipital lobe	13y3m	left	left posterior parietal and occipital lobectomy	right superior quadrantanopia	IID
UD	M	medically intractable focal epilepsy, symptomatic lesion/dysembryoplastic neuroepithelial tumor	abnormal MRI with lesion in the right temporal lobe	6y9m	right	right medial inferior temporal lobe gross total tumor resection with posterior temporal and occipital lobectomy	left hemianopia	IA
OT	M	medically intractable focal epilepsy, symptomatic lesion/ganglioglioma, FCD	abnormal MRI with lesion in the medial left temporal lobe	13y4m	left	medial left temporal lobe gross total tumor resection and left temporal lobectomy with preservation of medial structures	full	IA

* EEESOS: Engle Epilepsy Surgery Outcome Scale

Table S2. Patients' neuropsychological evaluation test performance pre- and post-surgery.

Code	Detailed IQ measures	Vision or visual motor integration	Memory & learning	Executive function	Academic skills/performance
KN	not done	not done	not done	not done	not done
SN	not done	not done	not done	not done	not done
TC	*Pre-surgery:*<br>WIAT III: 2nd percentile*<br>*Post-surgery:*<br>not done	not done	not done	not done	PPVT: 1st percentile*<br>WIAT III:<br>Reading: 1st grade*<br>Spelling: 2nd grade*
UD	*Pre-surgery:*<br>WASI: 116 (full scale), 135 (verbal), 97 (performance)<br>*Post-surgery:*<br>WASI: 118 (full scale), 123 (verbal), 108 (performance)	Grooved pegboard:<br>50th percentile<br>(dominant hand)	not done	Working memory<br>(from WISC-V):<br>*Post-surgery:*<br>34th percentile	WJ III ACH:<br>Reading: 63rd percentile<br>Letter-Word: 67th percentile<br>Passage: 56th percentile<br>Calculation: 91st percentile
OT	*Pre-surgery:*<br>WASI: 122 (full scale), 125 (verbal), 114 (performance)<br>*Post-surgery:*<br>WASI: 127 (full scale)	Grooved pegboard: average (dominant hand)	CVLT-C:<br>high average<br>WRAML-2:<br>high average	D-KEFS: superior	WJ III ACH: above age and grade expectancy

* Could not be reliably obtained due to language barrier
PPVT: Peabody Picture Vocabulary Test
CVLT-C: California Verbal Learning Test–Children's Version
D-KEFS: The Delis–Kaplan Executive Function System
Grooved Pegboard: Grooved Pegboard for Manipulation and Dexterity Testing
WASI: Wechsler Abbreviated Scale of Intelligence
WIAT-III: Wechsler Individual Achievement Test–Third Edition
WISC-V: Wechsler Intelligence Scale for Children–Fifth Edition
WJ III ACH: The Woodcock-Johnson III Tests of Achievement
WRAML-2: Wide Range Assessment of Memory and Learning–Second Edition

Table S3. Demographic information for each of the 25 fMRI controls.

Control	Gender	Age at scan	
		year	month
C1	F	7	1
C2	M	7	5
C3	M	8	6
C4	F	8	10
C5	M	8	11
C6	F	9	9
C7	M	10	3
C8	M	10	4
C9	F	10	8
C10	M	11	1
C11	M	11	5
C12	M	11	9
C13	M	12	1
C14	F	12	6
C15	M	12	10
C16	F	13	5
C17	M	14	4
C18	F	14	6
C19	F	14	9
C20	F	14	11
C21	M	15	0
C22	F	15	8
C23	M	16	3
C24	M	18	2
C25	M	18	8
